# The first case of a littoral spleen-preserving resection: a case report

**DOI:** 10.1093/jscr/rjad563

**Published:** 2023-10-20

**Authors:** Chen Lew, Sunny Dhadlie, Daniel Hussey, Naveen Mayavel, Stewart Skinner, Kasmira Wilson

**Affiliations:** Department of General Surgery, The Alfred Hospital, Melbourne, VIC 3004, Australia; Department of General Surgery, The Alfred Hospital, Melbourne, VIC 3004, Australia; Department of Anatomical Pathology, The Alfred Hospital, Melbourne, VIC 3004, Australia; Department of General Surgery, The Alfred Hospital, Melbourne, VIC 3004, Australia; Department of General Surgery, The Alfred Hospital, Melbourne, VIC 3004, Australia; Department of General Surgery, The Alfred Hospital, Melbourne, VIC 3004, Australia

**Keywords:** littoral cell angioma, spleen preserving, splenic mass, solitary lesion

## Abstract

Littoral cell angiomas are uncommon primary splenic haemangiomas with rare malignant potential. We report a case of a 76-year-old male with an incidental solitary littoral cell angioma found within an accessory spleen. We provide an overview of the literature of littoral cell angiomas and highlight the diagnostic challenge and treatment of this important differential for general surgeons caring for patients with splenic masses. This is the first case to describe primary resection of a littoral cell angioma with splenic preservation.

## Introduction

Littoral cell angioma’s (LCA) are rare primary splenic haemangiomas, with only several hundred cases described in literature to date [1]. These tumours originate from littoral cells lining splenic red pulp venous sinuses and possess malignant potential [1, 2]. Due to their asymptomatic nature, diagnosis is typically incidental. The ‘gold standard’ diagnostic tool for LCAs is histological diagnosis after excision [2, 3]. LCAs are often multifocal within the spleen and localized tumours are rare, thereby making splenectomy the mainstay of treatment [2, 4]. Here we present an incidental solitary LCA in an accessory spleen, making this the first case report describing primary resection of this tumour with splenic preservation.

## Case report

An otherwise well 76-year-old male presented after a high-speed motor vehicle accident. Contrast-enhanced computed tomography (CT) chest abdomen pelvis revealed an incidental solid left upper quadrant mass anterior to the spleen (49 × 39 mm^2^), and a left adrenal lesion. No splenic lesions were identified. History was significant only for intermittent episodes of self-limiting pain in the left upper quadrant. Subsequent investigation with quad-phase adrenal CT confirmed an adrenal adenoma but was unable to further characterize the left upper quadrant tumour. Tumour markers (alpha-fetoprotein, carbohydrate antigen-125, carbohydrate antigen-19-9 and Carcinoembryonic antigen) were within normal limits.

Ultrasound-guided core biopsy was non-diagnostic with histopathology demonstrating mixed inflammatory infiltrate on a background of fat necrosis. Biopsy was negative for gastrointestinal stromal tumour and vascular markers.

**Figure 1:**
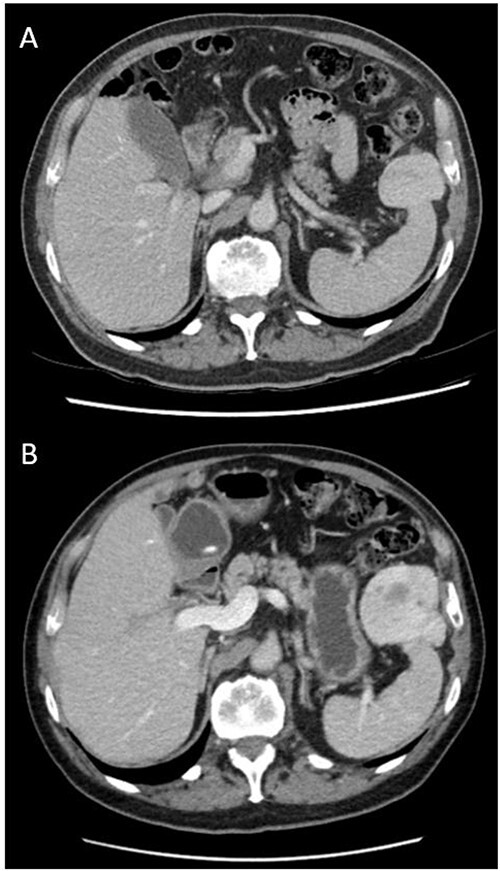
Axial views and comparison of original CT abdomen pelvis to 18-months later. (A) Contrast-enhanced CT abdomen pelvis (arterial phase): axial slice showing solid mass laterally in the left upper quadrant adjacent to the spleen measuring 49 × 39 mm^2^. The spleen is otherwise normal (B) Contrast-enhanced CT abdomen pelvis 18-months after original (arterial phase): Axial slice showing larger mass measuring 64 × 54 × 86 mm^3^ with persistent heterogeneous enhancement and prominent central area of decreased attenuation and peripheral cystic changes.

He was initially managed conservatively with surveillance imaging. Repeat CT abdomen pelvis 18 months after original imaging revealed an interval increase in the peri-splenic tumour (64 × 54 × 86 mm^3^), displaying a more prominent central area of decreased attenuation and peripheral cystic changes (Fig. 1). The lesion was abutting the anterior aspect of spleen and adjacent diaphragm. No abdominal lymphadenopathy was noted. Gastroscopy and colonoscopy were performed, confirming an extraluminal lesion. Due to continued diagnostic uncertainty, diagnostic laparotomy and excisional biopsy was performed.

**Figure 2:**
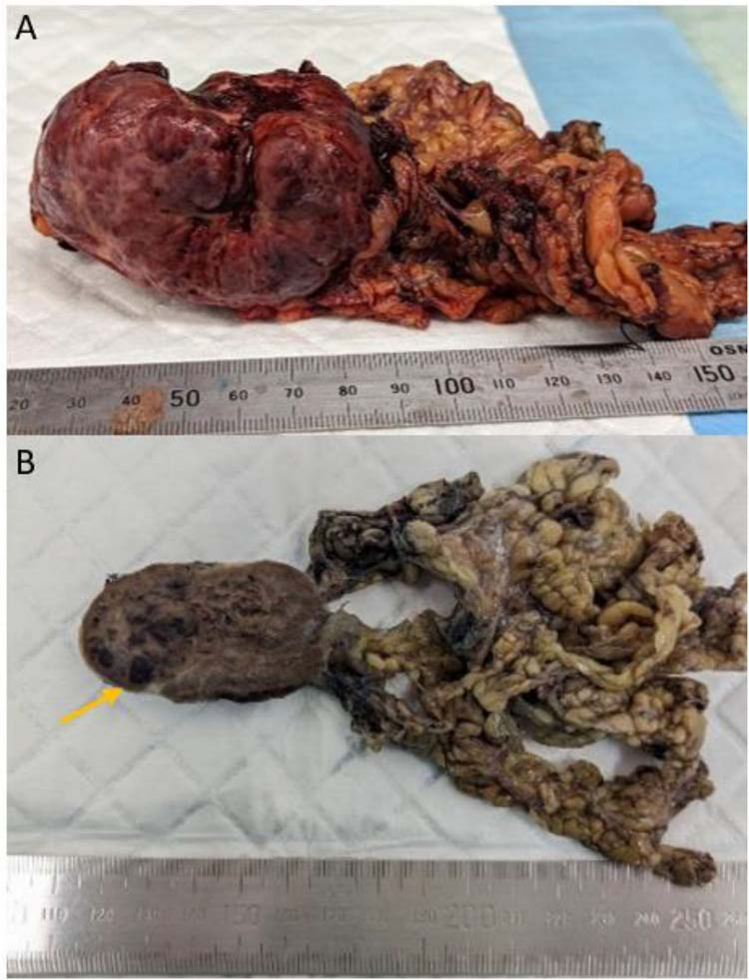
Gross appearance of the tumour consistent with littoral cell angioma. (A) Fresh pathology specimen of singular tumour from left upper quadrant with omentum attached, estimated length: 70 mm. (B) Specimen post fixation of cut surface, evidence of pools of blood (arrow).

Laparotomy revealed a vascular mass with a thin attachment to anterior aspect of spleen, and adhesions to the greater curvature of the stomach and splenic flexure. A spleen-preserving approach was undertaken with sharp dissection of the mass from the spleen (Fig. 2). The patient recovered well postoperatively without complication and was discharged home.

**Figure 3:**
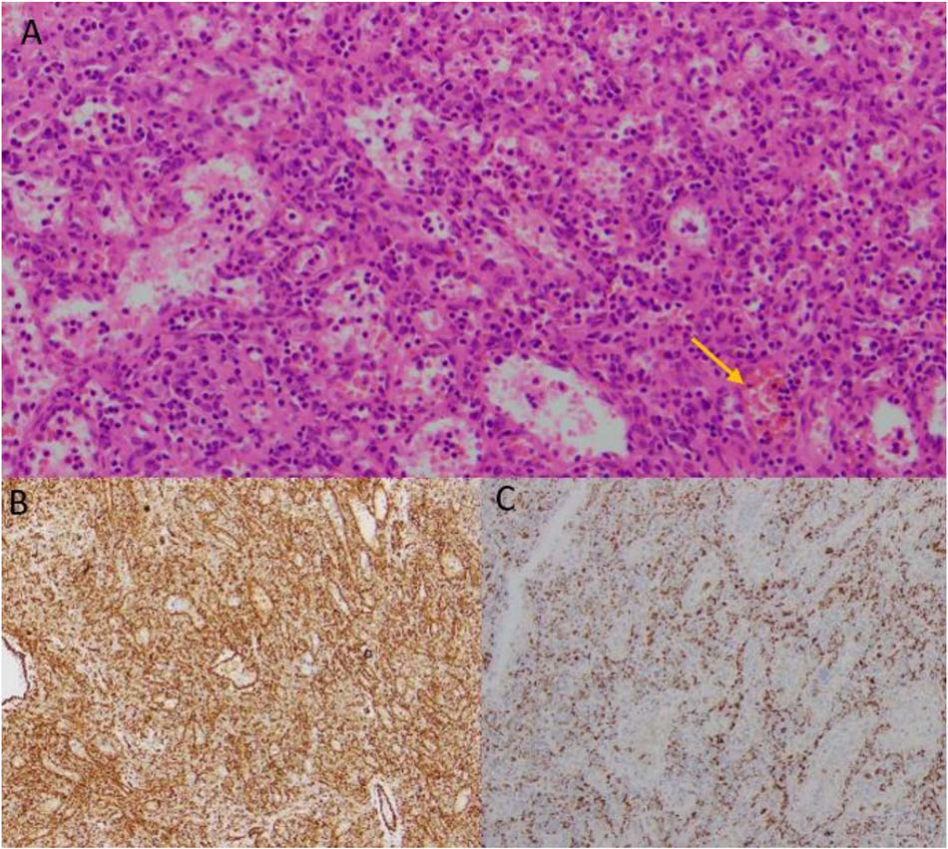
Histological images of the resected mass, showing evidence of littoral cell angioma. (A) Histology slide x200 magnification: multiple vascular channels lined by cells with reactive endothelium. There is focal haemorrhage (arrow). (B) Histology slide x100 magnification: staining positive for CD31. (C) Histology slide x200 magnification: staining positive for CD68.

Histology identified a benign littoral cell angioma, positive for endothelial markers (CD31 + CD34) and CD68 (histiocyte marker). Histopathology revealed interspersed nucleated red cells with occasional megakaryocyte-like cells and haemosiderin macrophages, indicative of extramedullary haemopoiesis (Fig. 3). The specimen weighed roughly 230 g. Histological results were reviewed in a multidisciplinary team meeting, with recommendation for ongoing surgical surveillance.

## Discussion

LCAs remain a diagnostic challenge, and largely rely on tissue diagnosis. Radiological imaging studies such as magnetic resonance imaging and CT have difficulty differentiating LCAs from other splenic tumours such as lymphoma, angiosarcomas and tumour metastasis [3, 5]. For LCAs, CTs are the mainstay of radiological evaluation, commonly showing multiple splenic masses <6 cm in diameter without associated lymphadenopathy [3, 5]. These vary in shape and typically have distinct but ill-defined margins. In our case, core biopsy was unable to provide a probable diagnosis however, Anbardar *et al.* suggests that fine-needle aspiration (FNA) may aid in diagnosis [6]. This is based on their review describing five cases of FNAs performed on LCAs that showed a low nuclear cytoplasmic ratio, tall cuboidal to columnar shaped cells, evenly distributed chromatin and indistinct nucleoli along with prominent intracytoplasmic hemosiderin pigment [6]. However, given the risk of bleeding of LCAs and location commonly within the spleen, FNA is not always feasible [1, 6–8].

Literature estimates that only 7-24% of LCAs present as solitary singular lesions, with most cases presenting with multifocal splenic nodules [2, 5, 9]. However, all cases of singular lesions in literature are contained within the spleen and are not amenable to isolated excision, making this case unique [2, 5, 9]. Literature suggests that current management of this condition is splenectomy, given the multifocal nature of LCA and potential for malignancy [4, 10]. Whilst few cases of LCAs are malignant (littoral cell angiosarcoma and littoral cell hemangioendothelioma), a 2022 review by Wang *et al.* identified 13.8% of LCAs were associated with other malignancies, most commonly lymphoma [2, 4]. No concurrent malignancy was diagnosed in our case. Wang *et al.* also highlights that risk of splenectomy may outweigh the risk of malignancy [2]. While 1.7% of patients in this review developed recurrence or metastasis post-splenectomy, no cause of death was confirmed to be directly related to the LCA while splenectomy had a post-operative mortality of 2.3% [2]. Furthermore, there have only been four documented cases of LCAs arising from an accessory spleen, which are congenital nodules of normal splenic tissue found apart from the main splenic body [3, 11–14]. However, all four cases had accompanying multifocal lesions within the spleen, and one case showed a recurrence in an unresected accessory spleen [11–14]. Our case is the first to describe a solitary LCA within an accessory spleen. Therefore, it is unknown whether there can be success with isolated excision of this tumour without splenectomy.

A review of literature has revealed only one documented case of an LCA excision without full splenectomy, in an 18-year-old female undergoing a partial splenectomy in 2012 [15]. In this case, partial splenectomy was able to be performed due to a solitary and localized LCA in the lower pole of the spleen [15]. No subsequent recurrence has been documented in this case. In the case presented here, the LCA was completely excised without any splenic tissue on histology. Current evidence suggests that splenectomy in patients with confirmed diagnoses of LCA may not be warranted, due to the high postoperative complication risk and low rate of metastasis in LCA, and LCAs may only require close follow-up instead [2]. However, there are minimal reports investigating follow-up of asymptomatic LCA, and further research is warranted to investigate this further.

Littoral cell angiomas are a rare but important differential for left upper quadrant masses. Current recommended treatment is aimed towards surgical excision with splenectomy. This report encompasses several key features of LCA management and is the first to report a successful spleen-preserving littoral cell angioma excision.

## Data Availability

All data supporting the findings of this study are included within the manuscript. No additional datasets or supplementary materials are required for a complete understanding of the research presented.
